# Antibody Responses after Classroom Exposure to Teacher with Coronavirus Disease, March 2020

**DOI:** 10.3201/eid2609.201802

**Published:** 2020-09

**Authors:** Nicole E. Brown, Jonathan Bryant-Genevier, Uptala Bandy, Carol A. Browning, Abby L. Berns, Mary Dott, Michael Gosciminski, Sandra N. Lester, Ruth Link-Gelles, Daniela N. Quilliam, James Sejvar, Natalie J. Thornburg, Bernard J. Wolff, John Watson

**Affiliations:** Centers for Disease Control and Prevention, Atlanta, Georgia, USA (N.E. Brown, J. Bryant-Genevier, M. Dott, S.N. Lester, R. Link-Gelles, J. Sejvar, N.J. Thornburg, B.J. Wolff, J. Watson);; Rhode Island Department of Health, Providence, Rhode Island, USA (U. Bandy, C.A. Browning, A.L. Berns, M. Gosciminski, D.N. Quilliam);; US Public Health Service Commissioned Corps, Rockville, Maryland, USA (M. Dott, R. Link-Gelles, J. Sejvar)

**Keywords:** 2019 novel coronavirus disease, coronavirus disease, COVID-19, severe acute respiratory syndrome coronavirus 2, SARS-CoV-2, viruses, respiratory infections, zoonoses, classroom, teacher, adolescents, United States

## Abstract

After returning from Europe to the United States, on March 1, 2020, a symptomatic teacher received positive test results for severe acute respiratory syndrome coronavirus 2. Of the 21 students exposed to the teacher in the classroom, serologic results suggested past infection for 2. Classroom contact may result in virus transmission.

In late February 2020, a teacher experienced headache, sore throat, myalgia, and fatigue while traveling in Europe, where community transmission of severe acute respiratory syndrome coronavirus 2 (SARS-CoV-2) was ongoing (*1*). After arriving back in the United States, the teacher returned to school February 24–27 while experiencing the same symptoms plus limited cough. An oropharyngeal swab sample collected on March 1 was positive for SARS-CoV-2 by reverse transcription PCR (cycle threshold values N1 = 35.05, N2 = 35.2; RNase P = 23.58). All students who attended classes with the infected teacher were instructed to quarantine themselves at home through March 12. After the quarantine period, we conducted a serologic survey to assess potential SARS-CoV-2 transmission in a classroom setting.

During February 24–27, the teacher taught 16 classes, all in the same room, each with <30 students. Of the 16 classes, 10 were discussion-based, in which the teacher reported walking around the room and speaking directly with students (interactive classes). For the other 6 classes, the teacher sat mostly in 1 location and close interactions with students were limited (noninteractive classes). On March 10, we contacted 120 students (48 [40%] enrolled in interactive classes, 72 [60%] enrolled in noninteractive classes) whose only known exposure was through classroom contact with the teacher and invited them to participate in our serologic survey; 21 (18%) students volunteered. 

Median participant age was 17 years (range 5–18 years). Five (24%) participants had interactive classroom contact; mean in-class time was 108 minutes. Sixteen (76%) participants had noninteractive classroom contact only; mean in-class time was 50 minutes.

Participating students completed a questionnaire about symptoms experienced during the quarantine period and provided a blood specimen. On March 13, whole blood (3–5 mL) was collected and serum was separated before samples were frozen at −80°C for shipping. The Centers for Disease Control and Prevention tested the samples for antibodies by ELISA, as described previously (B. Freeman et al., unpub. data, https://www.biorxiv.org/content/10.1101/2020.04.24.057323v2). We considered reciprocal titers of >400 to be positive and reciprocal titers of >100 but <400 to be indeterminate.

Of the 5 students with interactive classroom contact, results for 2 (students A and B) were suggestive of previous SARS-CoV-2 infection; results for student A were positive and for student B indeterminate (Table). Students A and B were not in the classroom during the same period and sat in different locations in the classroom. Student A had a reciprocal titer of 400 and spent 135 minutes in interactive classes. Beginning February 26, this student experienced intermittent myalgia, rhinorrhea, and cough for 9 days. Student B had a reciprocal titer of 100, spent 90 minutes in the interactive classroom, and reported no symptoms. The remaining 3 students (students C–E) had reciprocal titers of <100. Student C spent 135 minutes in interactive classes and reported no symptoms. Students D and E each spent 90 minutes in interactive classes and reported limited symptoms. Student D reported subjective fever and headache lasting 1 day, and student E reported rhinorrhea lasting 1 day. Although no serologic evidence of previous infection was found for participants with noninteractive classroom contact only, 7 (44%) reported symptoms. The most common symptoms among participants with noninteractive classroom contact were sore throat (n = 3), headache (n = 3), rhinorrhea (n = 2), and myalgia (n = 2).

**Table:** Antibody responses, classroom time, and symptoms experienced among students who had had interactive classroom contact with a teacher with confirmed coronavirus disease, March 2020

Student	ELISA result reciprocal titer	ELISA result interpretation	Minutes spent in interactive classroom	Symptoms (duration, d)
A	400	Positive	135	Myalgia (1), rhinorrhea (1), cough (3)
B	100	Indeterminate	90	None
C	<100	Negative	135	None
D	<100	Negative	90	Subjective fever (1). headache (1)
E	<100	Negative	90	Rhinorrhea (1)

Although SARS-CoV-2 transmission from symptomatic persons to close contacts has been well established, risks associated with classroom contact are not well known. The positive results for student A suggest past infection with SARS-CoV-2. The meaning of the indeterminate result for student B is less clear in this context, but this result may be suggestive of past infection. We do not know whether results from student A or B are indicative of immunity to SARS-CoV-2 infection. The symptoms reported by both students are consistent with those reported by children and adolescents with mildly symptomatic and asymptomatic coronavirus disease (*2**,**3*).

This survey is subject to limitations. First, we based the definition of interactive classroom contact on reported usual behavior by the teacher; however, variability of contact for each participant was not defined. Second, reported symptoms might have been affected by students’ expectation of the survey’s intent, leading to social desirability bias. Third, because of low participation, the results may not be generalizable to all students who had contact with the teacher. Fourth, among students who chose to participate, participation might have been influenced by their perceived risk or symptoms experienced during the quarantine period, leading to selection bias. Fifth, potential infections may not have been detected because blood collection ≈14 days after exposure may have been too soon for development of SARS-CoV-2 antibodies. Last, the only known exposure for participating students was the infected teacher; however, students could have been exposed by unrecognized community transmission. The risk for transmission from mildly symptomatic or asymptomatic persons is not well known.

Widespread school closures have mostly eliminated the risk for classroom transmission of SARS-CoV-2. However, these results suggest that classroom interaction between an infected teacher and students might result in virus transmission.
